# Adjunctive use of celecoxib with anti-tuberculosis drugs: evaluation in a whole-blood bactericidal activity model

**DOI:** 10.1038/s41598-018-31590-4

**Published:** 2018-09-10

**Authors:** Claire M. Naftalin, Rupangi Verma, Meera Gurumurthy, Kim Hor Hee, Qingshu Lu, Benjamin Chaik Meng Yeo, Kin Hup Tan, Wenwei Lin, Buduo Yu, Kok Yong Seng, Lawrence Soon-U Lee, Nicholas I. Paton

**Affiliations:** 1 Department of Medicine, Yong Loo Lin School of Medicine, National University of Singapore, Singapore, Singapore; 2 Singapore Clinical Research Institute, Singapore, Singapore; 3 Investigational Medicine Unit, National University Health System, Singapore, Singapore

## Abstract

COX-2 inhibition may be of benefit in the treatment of tuberculosis (TB) through a number of pathways including efflux pump inhibition (increasing intracellular TB drug levels) and diverse effects on inflammation and the immune response. We investigated celecoxib (a COX-2 inhibitor) alone and with standard anti-tuberculosis drugs in the whole-blood bactericidal activity (WBA) model. Healthy volunteers took a single dose of celecoxib (400 mg), followed (after 1 week) by a single dose of either rifampicin (10 mg/kg) or pyrazinamide (25 mg/kg), followed (after 2 or 7 days respectively) by the same anti-tuberculosis drug with celecoxib. WBA was measured at intervals until 8 hours post-dose (by inoculating blood samples with *Mycobacterium tuberculosis* and estimating the change in bacterial colony forming units after 72 hours incubation). Celecoxib had no activity alone in the WBA assay (cumulative WBA over 8 hours post-dose: 0.03 ± 0.01ΔlogCFU, p = 1.00 versus zero). Celecoxib did not increase cumulative WBA of standard TB drugs (mean cumulative WBA −0.10 ± 0.13ΔlogCFU versus −0.10 ± 0.12ΔlogCFU for TB drugs alone versus TB drugs and celecoxib; mean difference −0.01, 95% CI −0.02 to 0.00; p = 0.16). The lack of benefit of celecoxib suggests that efflux pump inhibition or eicosanoid pathway-related responses are of limited importance in mycobacterial killing in the WBA assay.

## Introduction

There has been considerable recent interest in the use of adjunctive host-directed therapy to enhance activity of anti-tuberculosis drugs^1,2^. Drugs that inhibit cyclo-oxygenase (COX) enzymes have been identified as a class of potential value in tuberculosis (TB), with a number of actions that may be of putative benefit.

COX inhibitors may increase intracellular drug concentrations through COX-2 dependent regulation of human membrane-associated p-glycoprotein transporter (MDR-1), an efflux pump which is homologous to the bacterial MDR-1 transporter^3^. COX-2 inhibition has been reported to increase intracellular concentrations of certain drugs in the human host^4^ and increase the sensitivity of bacterial populations to antibiotics^3^. Rifampicin itself can lead to induction of efflux and transporter gene expression thereby reducing intra-bacterial drug levels^5^, and thus COX-2 inhibitor drugs may counteract this effect leading to enhanced efficacy of anti-tuberculous drugs.

These drugs also have diverse immunological and inflammatory effects that may affect the course of TB disease. Prostaglandin E2 (PGE2), one inflammatory mediator produced predominantly by COX-2^6^, appears to have complex pro- and anti-inflammatory roles in TB infection depending on its concentration and the stage of infection^7^. In established TB infection, increased PGE2 levels are associated with downregulation of Th1 response^8^ and macrophage deactivation^9^, possibly augmenting lung pathology^7^. Inhibition of COX activity reduces formation of PGE2 in response to inflammatory stimuli^10^. Dosing TB-infected murine models with COX inhibitors reduces lung infiltrates, lowers bacillary loads and improves survival^7,11–14^. COX suppression also inhibits immune activation of T cells by *Mycobacterium tuberculosis* (Mtb)^15^. In addition, synergy between anti-tuberculosis drugs and COX inhibition (pyrazinamide with ibuprofen^16^ and streptomycin with diclofenac^12^) has been seen in mouse models.

Celecoxib is a widely-used selective COX-2 inhibitor, reduces levels of PGE2^10,17^ and has anti-inflammatory properties effective in treating osteoarthritis, rheumatoid arthritis and acute pain^18^. Administration of celecoxib has shown benefit in mice infected with tuberculosis^13^.

Whole-blood bactericidal activity (WBA) is an *ex vivo* assay which has been used successfully to assess the effects of TB drugs in combination with the host immune response on mycobacterial killing^19,20^. WBA is effectively able to directly evaluate the immune response of the host^21,22^, with reports of significant inhibition of mycobacterial growth following BCG vaccination in the absence of anti-mycobacterial drugs^22^. We hypothesized that celecoxib, either through efflux pump inhibition or through direct immunological effects, might reduce mycobacterial growth in a WBA assay, and tested this in a healthy-volunteer clinical trial.

## Results

### Participants

A total of 21 volunteers were screened, of whom 2 were excluded due to abnormal screening blood results, and 1 withdrew prior to randomization. The 18 randomised (9 to rifampicin (RIF) and 9 to pyrazinamide (PZA) group) were all male; median age of 30 years old (range 24–39); median weight 73.7 kg (range 56.2–98.6); 16 Chinese, 2 Indian; all HIV negative; 2 IGRA positive.

Two participants withdrew after completion of the second visit (both in RIF group) reporting minor clinical adverse events (nasal congestion, febrile illness). Data up to the point of withdrawal are included in the analysis. There were 4 adverse events, all grade 1 (nasal congestion and febrile illness in the RIF group; elevated alanine transaminase and hypokalaemia in the PZA group).

### Effect of celecoxib on anti-mycobacterial activity in WBA model

**Figure 1:**
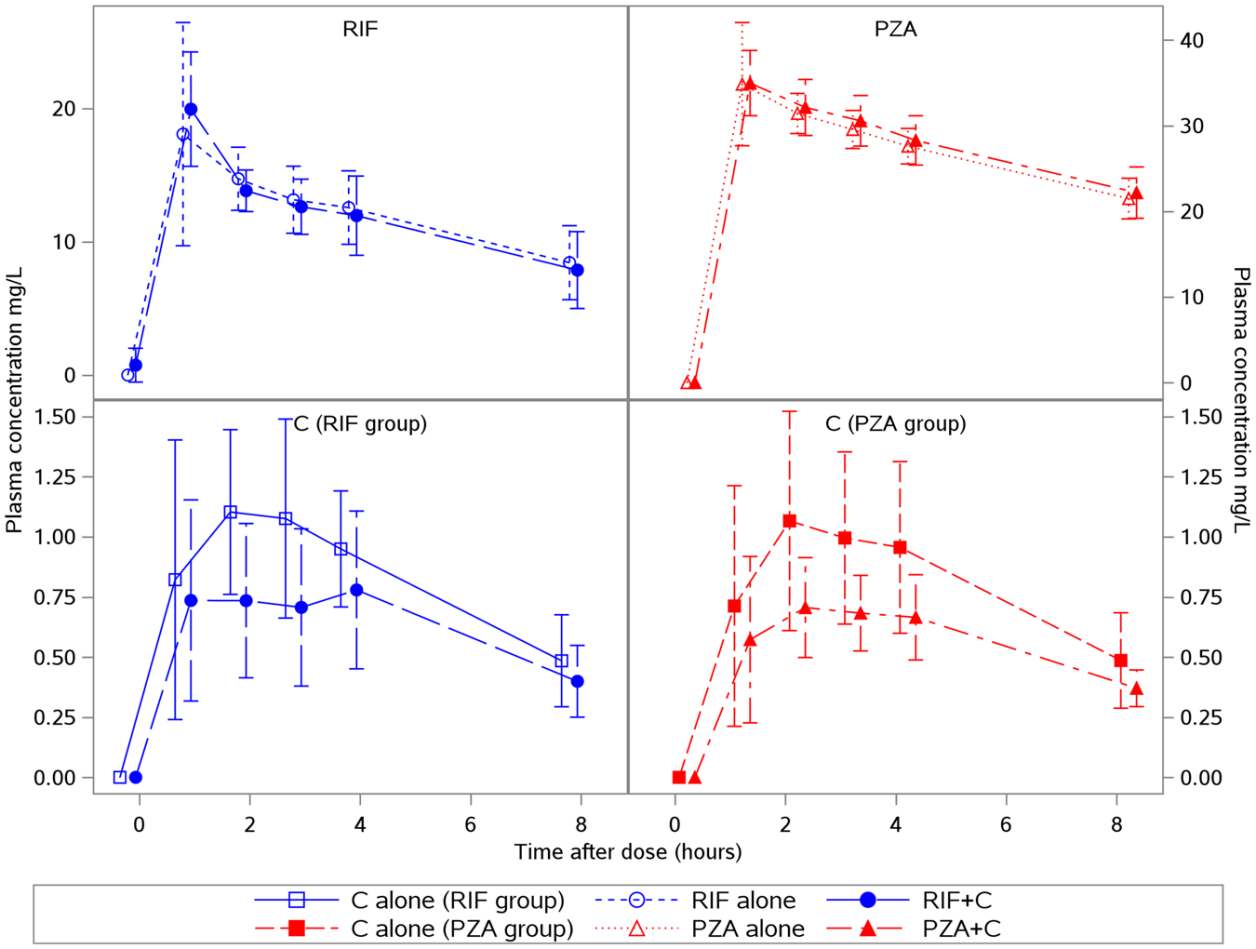
Plasma drug concentration of rifampicin (upper left panel), pyrazinamide (upper right panel), celecoxib in the RIF group (lower left panel), and celecoxib in the PZA group (lower right panel) at individual time-points up to 8 hours after dose. On each panel plots of plasma concentration are separate for two study visits where a drug or its combination with another drug is administered. Abbreviations: C, celecoxib; RIF, rifampicin; PZA, pyrazinamide.

**Figure 2:**
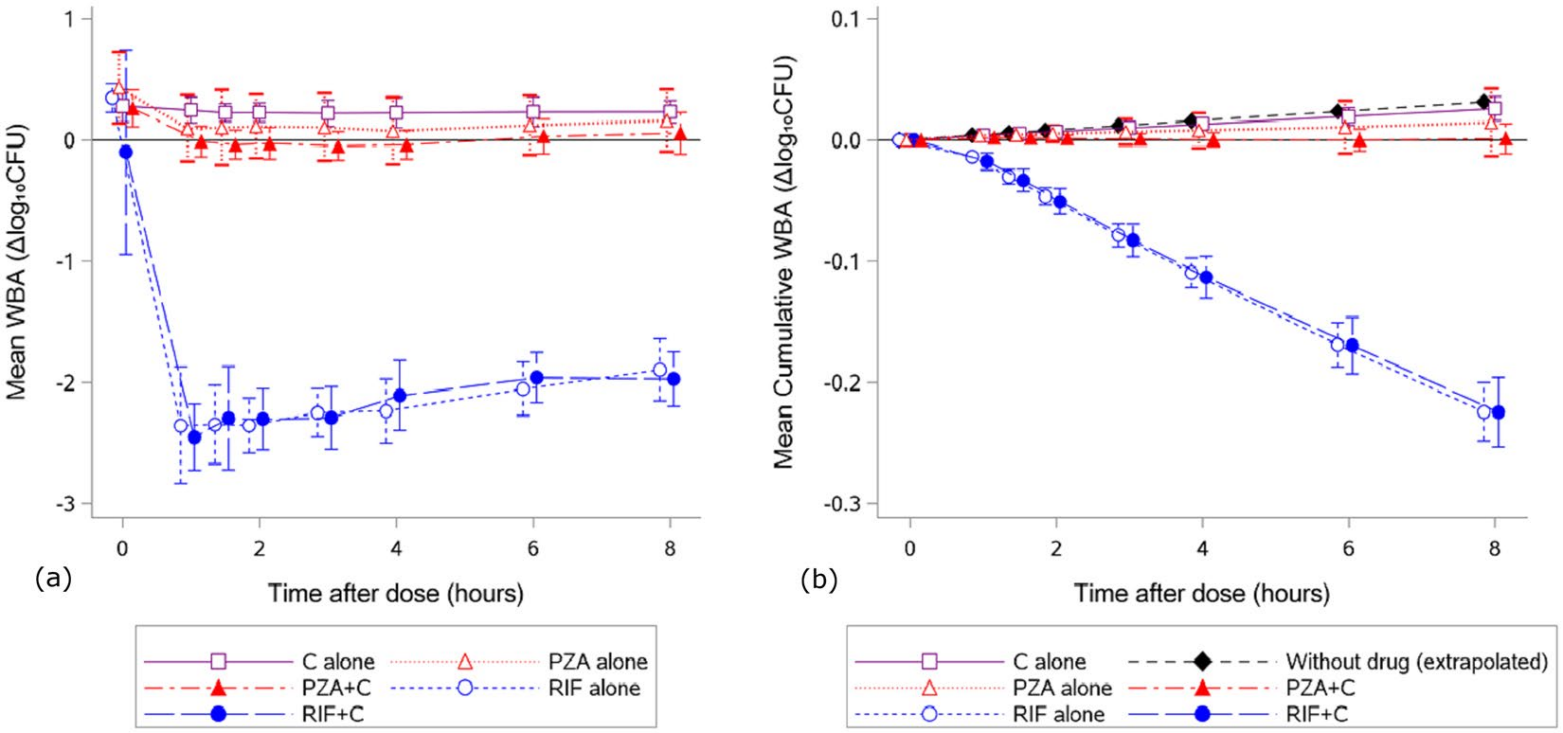
(**a**) Mean WBA at individual time-points up to 8 hours after dose. (**b**) Mean cumulative WBA at intervals up to 8 hours post-dose. The curve of “Without drug (extrapolated)” was obtained by assuming that the individual WBA values at 0 hour time-point (pre-dose) of celecoxib alone remain unchanged over a subsequent 8-hour interval. Abbreviations: C, celecoxib; RIF, rifampicin; PZA, pyrazinamide.

There was no significant effect of celecoxib on rifampicin or pyrazinamide drug levels, although there was a non-significant trend towards lower C_max_ and AUC_(0–8hrs)_ of celecoxib when co-administered with the TB drugs (Table 1, Fig. 1). Celecoxib alone had no bactericidal activity (mean WBA values above zero at all individual time-points throughout the 8 hour study period; p = 1.00 versus zero for both maximum WBA (WBA_max_) and cumulative WBA (WBA_cum_); Table 2, Fig. 2). Rifampicin alone showed strong bactericidal activity over 8 hours (WBA_max_ −2.48 ± 0.24ΔlogCFU, p < 0.0001 versus zero; WBA_cum_ −0.22 ± 0.02ΔlogCFU, p < 0.0001 versus zero; Table 2, Fig. 2). Pyrazinamide showed no bactericidal activity (WBA_max_ −0.03 ± 0.23ΔlogCFU, p = 0.37 versus zero; WBA_cum_ 0.01 ± 0.03ΔlogCFU, p = 0.92 versus zero; Table 2, Fig. 2), although it appeared to be bacteriostatic (Fig. 2b).

**Table 1:** Summary of Pharmacokinetic (PK) Characteristics.

	Celecoxib levels			P^a^	Rifampicin levels		P^a^	Pyrazinamide levels		P^a^
	C alone	RIF + C	PZA + C		RIF alone	RIF + C		PZA alone	PZA + C	
AUC_(0–8)_, h*mg/L	5.97 (2.91, 10.25)	4.53 (1.50, 6.43)	4.23 (2.04, 6.34)	0.07	92.26 (72.59, 137.22)	90.80 (71.88, 136.02)	0.76	207.36 (182.89, 236.74)	211.92 (183.50, 249.57)	0.36
C_max_, mg/L	1.14 (0.49, 2.02)	0.89 (0.29, 1.27)	0.76 (0.37, 1.38)	0.08	19.31 (12.63, 30.98)	19.53 (13.43, 26.53)	0.94	35.50 (29.73, 48.10)	35.14 (30.28, 40.30)	0.79
T_max_, h	2.0 (1.0, 8.0)	2.0 (1.0, 4.0)	3.0 (1.0, 4.0)	0.93	1.0 (1.0, 3.0)	1.0 (1.0, 1.0)	0.50	1.0 (1.0, 2.0)	1.0 (1.0, 2.0)	1.00
t_1/2_^b^, h	4.48 (1.48)	6.65 (2.62)	7.28 (5.84)	0.10	8.04 (3.33)	7.70 (4.07)	0.27	11.45 (3.50)	11.24 (2.34)	0.83

Values are geometric mean and range (min, max) for AUC_(0–8)_ and C_max_; median (min, max) for T_max_; mean (SD) for t_1/2_.

^a^P values for comparison of PK parameters AUC_(0–8)_ (log-transformed), C_max_ (log-transformed) and t_1/2_ between groups by repeated-measure ANOVA (celecoxib levels) or paired-sample t-test (rifampicin levels and pyrazinamide levels), and T_max_ by Friedman’s test (celecoxib levels) or Wilcoxon signed rank test (rifampicin levels and pyrazinamide levels).

^b^t_1/2_ estimation includes 24 hour data where available (6 in PZA group, 2 in RIF group).

Abbreviations: C, celecoxib; RIF, rifampicin; PZA, pyrazinamide.

**Table 2:** Whole-blood bactericidal activity (WBA) results.

	Celecoxib alone	TB drug alone	TB drug + Celecoxib	Mean difference^a^ (95% CI)	P value^b^
TB drugs (RIF + PZA groups combined)	n = 18	n = 18	n = 16		
WBA_max_ (ΔlogCFU)	0.14 (0.10)	−1.25 (1.28)	−1.16 (1.24)	−0.07 (−0.17, 0.04)	0.20
WBA_cum(0–8)_ (ΔlogCFU)	0.03 (0.01)	−0.10 (0.13)	−0.10 (0.12)	−0.01 (−0.02, 0.00)	0.16
**RIF group**	**n** = **9**	**n** = **9**	**n** = **7**		
WBA_max_ (ΔlogCFU)	0.17 (0.09)	−2.48 (0.24)	−2.51 (0.23)	−0.03 (−0.25, 0.18)	0.72
WBA_cum(0–8)_ (ΔlogCFU)	0.03 (0.01)	−0.22 (0.02)	−0.22 (0.03)	0.00 (−0.02, 0.02)	0.96
**PZA group**	**n** = **9**	**n** = **9**	**n** = **9**		
WBA_max_ (ΔlogCFU)	0.12 (0.10)	−0.03 (0.23)	−0.12 (0.10)	−0.09 (−0.22, 0.04)	0.14
WBA_cum(0–8)_ (ΔlogCFU)	0.02 (0.01)	0.01 (0.03)	0.00 (0.01)	−0.01 (−0.03, 0.00)	0.09

Values are mean (SD), and mean difference with 95% CI.

^a^Mean difference for TB drug with and without celecoxib is derived from those with paired data for the TB drug i.e. n = 16 for combined groups, n = 7 for RIF group, n = 9 for PZA group.

^b^WBA values were compared between TB drug alone and TB drug plus celecoxib using a paired-sample t-test.

P values for WBA_max_ and WBA_cum_ versus zero: 1.00 and 1.00 (Celecoxib alone); <0.0001 and <0.0001 (RIF alone); 0.37 and 0.92 (PZA alone).

Abbreviations: RIF, rifampicin; PZA, pyrazinamide; CI, confidence interval.

In the analysis with TB drug groups combined, addition of celecoxib did not increase either WBA_max_ (difference −0.07, 95% CI −0.17 to 0.04; p = 0.20) or WBA_cum_ (difference −0.01, 95% CI −0.02 to 0.00; p = 0.16) (Table 2). There was also no significant effect of celecoxib on WBA_max_ or WBA_cum_ when the two drug groups were analysed separately (all p ≥ 0.09; Table 2, Fig. 2).

**Figure 3:**
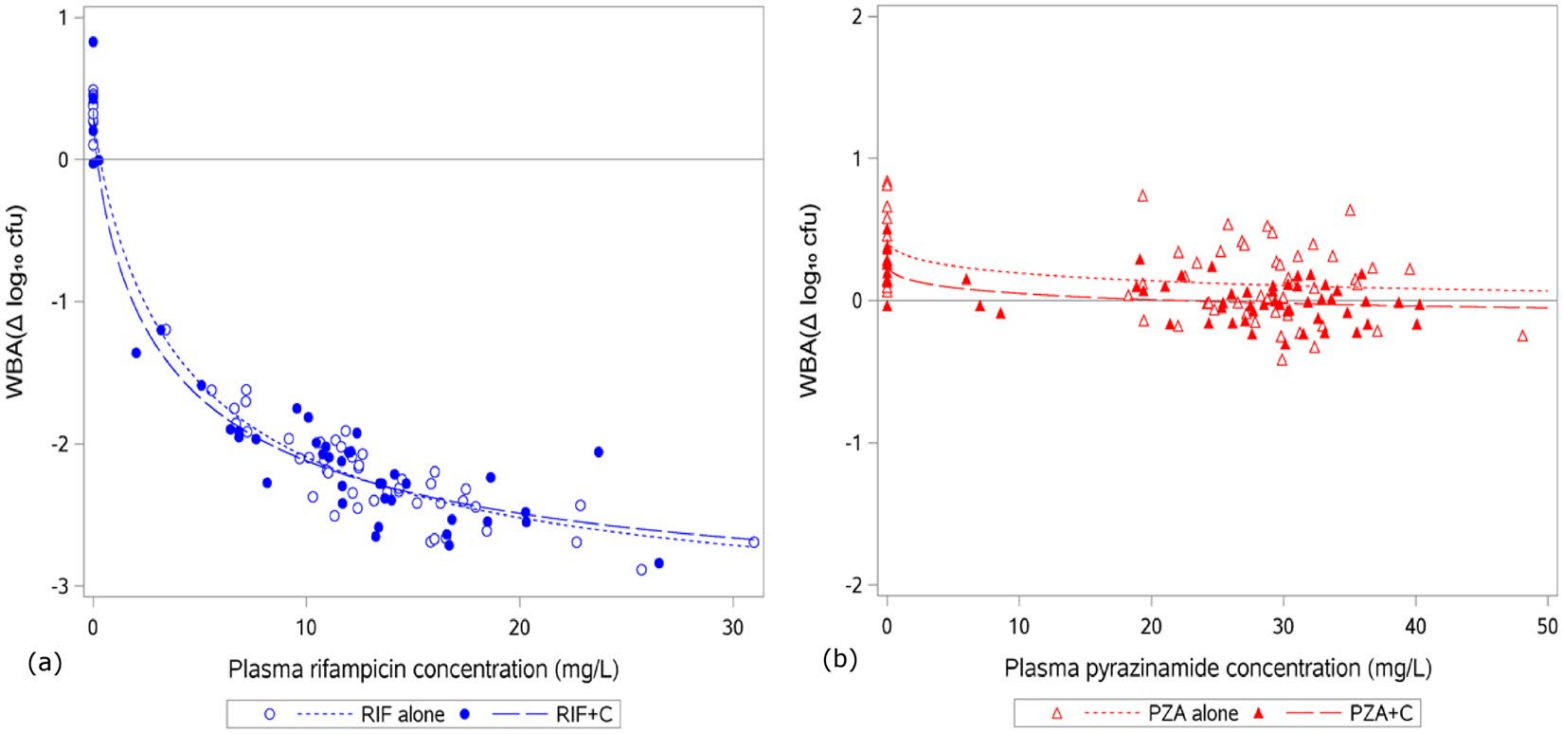
Relationship between plasma concentration of TB drug and WBA. Each point represents an individual blood sample at which both parameters were measured. (**a**) Rifampicin plasma concentration and WBA. The fitted model between WBA (*y*) and pyrazinamide concentration (*x*) was $y=0.35-3.64\frac{{(x/4.42)}^{0.87}}{1+{(x/4.42)}^{0.87}\,}$ in RIF alone and $y=0.33-3.60\frac{{(x/3.71)}^{0.76}}{1+{(x/3.71)}^{0.76}\,}$ in RIF+ C (p = 0.80 for comparison of model between RIF alone and RIF+ C). (**b**) Pyrazinamide plasma concentration and WBA. The fitted model between WBA (*y*) and pyrazinamide concentration (*x*) was $y=0.43-0.59\frac{{(x/19.94)}^{0.54}}{1+{(x/19.94)}^{0.54}\,}$ in PZA alone and $y=0.26-0.51\frac{{(x/19.88)}^{0.50}}{1+{(x/19.88)}^{0.50}\,}$ in PZA+C (p = 0.57 for comparison of model between PZA alone and PZA+ C). Abbreviations: C, celecoxib; RIF, rifampicin; PZA, pyrazinamide.

Bactericidal activity was significantly higher with increasing plasma concentrations of RIF (p < 0.0001) and PZA (p = 0.001), but the WBA response versus drug concentration curve was no different for the same plasma concentration of either TB drug when co-administered with celecoxib (p = 0.80 for RIF and p = 0.57 for PZA) (Fig. 3).

## Discussion

We found no evidence of bactericidal activity of celecoxib alone, or when used in combination with rifampicin or pyrazinamide in the WBA model. This does not support our hypothesis that celecoxib would increase WBA killing through blocking the efflux pump (and thereby increase intracellular drug concentrations) in bacteria (and/or host)^3,4^. In contrast, other bacterial efflux pump inhibitors, such as verapamil and reserpine, have been shown to increase susceptibility to TB drugs (although this has not been tested in the WBA model)^23,24^. Efflux pathway inhibition might be expected to be of particular benefit with pyrazinamide, the action of which depends on the intra-bacterial conversion of pyrazinamide to pyrazinoic acid; inhibition of the bacterial pump might prevent the active metabolite from diffusing out of the bacteria^23^. This effect would have been expected to be of relatively rapid onset, detectable within the time frame of WBA assay incubation^3,4^. The absence of this effect may in part be attributable to the relative insensitivity of the WBA assay to the effects of PZA compared to other TB drugs. Previous studies of pyrazinamide have shown, as in this study, limited or no WBA efficacy of pyrazinamide when used alone^25,26^ (although a study with a sample size of 1 did suggest some effect^19^); and there was evidence of only modest WBA activity when pyrazinamide was used in combination with other anti-tuberculous drugs^20,26^. The limited effect of PZA *ex vivo* may possibly be explained by the absence, in the WBA assay, of the acidic environment considered essential for PZA-induced Mtb killing inside pulmonary lesions *in vivo*^27,28^. However, we did not see an additive effect of celecoxib with rifampicin either, even though rifampicin shows strong bactericidal activity in the WBA assay. In this case, the absence of additional effect of celecoxib may possibly reflect saturation of the assay by rifampicin. If this were the explanation, then performing the experiment using a rifampicin-resistant Mtb strain might be able to demonstrate better any increased bactericidal activity arising from increased intracellular drug levels consequent on efflux pump inhibition^5,29^.

We also hypothesised that celecoxib might affect growth in the WBA assay through the direct T-cell immunomodulatory effects of COX inhibition^15^. The *ex vivo* WBA paradigm has the potential advantage over *in vitro* assays of evaluating the integration of effects of anti-mycobacterial drugs with host immune responses, both of which have been established independently in healthy volunteers using the WBA model^19,22,26^. The patho-physiological mechanisms of response to TB infection in patients are complex, depending on multiple factors including pre-existing host immunity, burden of infection and individual inflammatory responses upon antigen exposure. Whilst acknowledging that the WBA paradigm has the innate limitations of all *ex vivo* models, we hypothesised that administering celecoxib to healthy individuals followed by stimulation of whole blood with Mtb might affect growth in the WBA assay through the direct T-cell effects of COX inhibition^15^. This immunological effect might be expected to be most clearly seen in the celecoxib alone group, without the confounding effects of other drugs. However, we observed little effect with celecoxib alone compared to projected bacterial growth over the time period of the assay. We did not look directly for immunological changes in our WBA assay, so cannot say whether the lack of growth inhibition is due to the absence of immunological changes with celecoxib, or the lack of an effect of those immunological changes on growth in the WBA paradigm. We can be confident that the single dose of celecoxib 400 mg inhibited COX-2 and reduced PGE2 levels in an *ex vivo* setting following stimulation^10,17^, but substantive effects on T-cell immune responses may need more sustained exposure. However, the WBA assay incubation should have been of sufficient duration for immunological changes to occur, as these were seen after 12 hours incubation with indomethacin (another COX inhibitor) in a previous *in vitro* study^15^. Ours is the first study that has attempted to use the WBA model to detect effects of host-directed therapy and it is possible that the model is simply not responsive to such interventions. We did this study in healthy volunteers rather than TB patients. Although this is unlikely to affect the assessment of drugs acting directly on bacteria (as above), there may be substantive differences between blood from healthy volunteers and TB patients in the immune and inflammatory responses following exposure to exogenous mycobacteria in the WBA assay. Further studies evaluating other immune-based interventions and done in both patients with TB and in healthy volunteers are required for a more comprehensive determination of the value of the WBA paradigm in the initial assessment of putative host-directed therapies.

Our findings also contrast with most animal model pre-clinical studies that suggest overall benefit of COX inhibition (mediated by reduction of PGE2 levels) in TB, either alone or in combination with anti-mycobacterial drugs^7,11–14^. However, the results are not totally consistent between these studies, likely explained by the diverse study designs and the distinct differences in the host immune response to Mtb infection between different mouse models. One mouse model study even shows the opposite effect, with enhanced mycobacterial clearance following intranasal administration of PGE2^30^, highlighting the complexity of using animal models to study host-directed therapy in TB. Variability in potency of COX receptor inhibition between the different study drugs used may also partially explain the contrast between our results and published literature^31^. We cannot rule out the possibility that other COX inhibitors with greater inhibition of PGE2 than celecoxib, such as diclofenac or etoricoxib^32^, might have had a more enhanced anti-mycobacterial effect, although we would have expected celecoxib to show some inhibition of Mtb growth if our postulated mechanism of action could be detected in the WBA model. Although plasma celecoxib levels appeared to be lower when co-administered with TB drugs, concentrations should have been sufficient (both when celecoxib was given alone and with TB drugs) to show an effect on WBA via the PGE2 pathway, if such an effect exists^33^. In these *in vivo* settings, PGE2 most likely exerts its effects primarily in tissue lesions (in the lung and elsewhere), and such effects could not be detected in a model with growth in whole blood. Furthermore, there are well-known differences between mouse and human immune response to TB, and so it is possible that the role and impact of the PGE2 pathway may differ between the species.

In summary, celecoxib alone or in combination with rifampicin or pyrazinamide did not have anti-mycobacterial effects in the WBA model in spite of several postulated mechanisms of action that might have been detected in this paradigm. *In vivo* clinical trials currently underway [NCT02503839; NCT02781909] may be better suited to explore the utility of adjunctive COX inhibition in combination with TB drugs.

## Methods

Healthy volunteers between 21 and 70 years old were identified from a volunteer database and recruited over a period of 4 months (Nov 2015 to Feb 2016). Exclusion criteria were pregnancy or breastfeeding; body weight below 50 kg; clinical evidence of active TB; known allergy to study drugs; use of study drugs or drugs with known interaction with study drugs or cytochrome P450 enzymes inducers or inhibitors; serum creatinine level or liver enzyme activity above upper limit of normal; gout or acute porphyria; hepatitis; alcohol abuse; other risk factors for cardiovascular events or gastrointestinal bleeding.

Participants attended 3 visits, fasted for at least 6 hours prior to each visit. At the 1^st^ visit, all participants took a single dose of celecoxib 400 mg (Lek, Slovenia). The single dose of celecoxib 400 mg was determined from pharmacokinetic data to simulate the steady state effect of celecoxib 200 mg BID^33^, which is the maximum recommended dose for the majority of clinical indications^18^. At the 2^nd^ visit (7 days later) participants took a single dose of either rifampicin 10 mg/kg (Remedica, Cyprus; dose rounded up to nearest 150 mg) or pyrazinamide 25 mg/kg (23.1–27.3 mg/kg) (Novartis, Bangladesh; dose administered according to standard weight bands^34^). Allocation to RIF or PZA groups was by randomisation in 1:1 ratio, performed using pre-prepared opaque envelopes containing study codes. At the 3^rd^ visit (2 days later in the RIF group, 7 days later in the PZA group) participants were given the TB drug together with celecoxib (same doses). Treatment was open label and all study drugs were administered orally.

Participants were asked about symptoms at each visit. HIV antibody and IGRA tests were done at the first visit and a full blood count (FBC), renal function and liver enzymes at 3^rd^ visit. At each study visit, blood was taken pre-dose and at 1, 1.5, 2, 3, 4, 6, 8 hours post-dose. WBA was measured at all time-points to 8 hours, and drug levels at pre-dose and at 1, 2, 3, 4 and 8 hours post-dose. An additional sample (optional) was taken for drug level measurement at 24 hours post-dose at 3^rd^ visit. Blood samples for WBA were kept at room temperature for a maximum of 8 hours prior to analysis; blood for drug level measurement was centrifuged within 30 minutes of collection and plasma was frozen at −20 °C.

### Pharmacokinetic assays

Celecoxib, rifampicin and pyrazinamide levels in plasma were measured using a modified LC-MS/MS method^35,36^. Celecoxib, rifampicin and pyrazinamide were purchased from Toronto Research Chemicals Inc. (Ontario, Canada). Calibration samples of celecoxib (0.1–20 µg/ml), rifampicin (0.25–50 µg/ml) and pyrazinamide (1–200 µg/ml) in plasma were prepared by adding stock solutions of the drugs (10 mg/ml) in blank plasma followed by serial dilution. Analytes were extracted from each sample by protein precipitation using methanol (containing stable isotope-labeled internal standards celecoxib-d4, rifampicin-d3 and pyrazinamide-15Nd3 at 5, 5 and 2 µg/ml) before injecting into the LC-MS/MS system. The LC-MS/MS system consisted of Agilent 1290 UHPLC equipped with a cooled autosampler (6 °C) connected to Agilent 6460 triple quadrupole mass spectrometer (Agilent Technologies, Wald-bronn, Germany). Chromatographic separations were achieved using Zorbax Eclipse Plus C18 column (Agilent, 50 mm × 2.1 mm, 1.8 µm) with gradient elution. The mass spectrometer was operated under positive ionization mode and the detection of celecoxib, rifampicin and pyrazinamide was based on multiple-reaction monitoring of 382 → 362, 823 → 791 and 124 → 81 m/z, respectively.

Pharmacokinetic (PK) parameters were estimated by non-compartmental analysis using WinNonlin software (version 6.4, Certara Inc., Princeton, NJ). The area under the plasma concentration versus time curve from 0 to 8 hours (AUC_(0–8)_) were calculated according to the linear up/log down trapezoidal rule. The terminal half-life (t_1/2_) in plasma was calculated from the elimination rate constant (k_e_), estimated as the slope of the log-linear terminal portion of the mean plasma concentration versus time curve, by linear regression analysis (using data from 24 hour post-dose measurements where available).

### Whole-blood bactericidal activity assay

The WBA assay methodology was adapted from previously published methods^19,20,37^. In brief, standard stock for all experiments was made using *Mycobacterium tuberculosis* (H37Rv) grown in 7H9 medium to mid-log phase and then frozen in 10% glycerol aliquots of OD 1.0 at −80 °C. A standard curve relating the time to positivity (TTP) in culture to volume of the stock was generated by performing serial 5-fold dilutions of stock (25 to 25 × 10^−8^ µL) in 7H9 medium in duplicates, inoculating into MGIT tubes and incubating in the MGIT960 detection system (Becton Dickinson, Franklin Lakes, USA).

The volume of mycobacterial stock calculated from the standard curve to give a TTP of 5.5 days (0.5 µL) was added to heparinized blood (300 µL), and topped up with sufficient tissue culture medium (RPMI-GlutaMAX) to make up total culture volume of 600 µL. Cultures were set-up in sealed screw-cap tubes and incubated at 37 °C with slow constant mixing for 72 hours. Following incubation, cultures were centrifuged at 12,000RPM for 5 minutes and the liquid phase was removed. Blood cells were lysed by adding 1 ml sterile water to the pellet and vortexing over 10 minutes. Samples were centrifuged again (12,000RPM) for 10 minutes and the supernatant discarded. The pellet was re-suspended in 500 µL of 7H9 medium and inoculated into MGIT tubes, and the TTP recorded (to the nearest minute). WBA cultures were set-up in duplicate at every time-point and the mean TTP calculated. Control cultures (in duplicate) were set-up on the same day by inoculating the standard volume of stock directly into MGIT tubes. The WBA at each of the individual sample time-points was obtained from the difference between the log of the volume on the standard curve that corresponded to the TTP for that time-point and the log of the volume corresponding to the TTP of the control culture. This is equivalent to the difference in log of bacterial colony forming units (CFU) between the sample and the control, reported as ΔlogCFU.

### Sample size and statistics

We calculated the sample size needed to detect a difference in cumulative WBA of 0.1ΔlogCFU between TB drug with and without celecoxib, assuming a standard deviation (SD) of 0.1ΔlogCFU based on previously published data^38^. We assumed the adjunctive effect of celecoxib would not differ according to the concomitant anti-mycobacterial drug. A total sample size of 18 participants gave a power of >90% to detect the specified difference of 0.1ΔlogCFU, with alpha 0.05 using a two-sided paired-sample t-test.

A two-sided paired-sample t-test (or repeated measured ANOVA for comparison of celecoxib levels) was used to compare PK parameters (C_max_, AUC_(0–8hrs)_[both log-transformed] and t_1/2_). T_max_ was compared by the Wilcoxon signed rank test (or the Friedman test for comparison of celecoxib levels). The WBA_max_ for each participant was calculated as the lowest WBA value at each study visit up to 8 hours (positive WBA values indicate bacterial growth and negative values killing). WBA_cum_, the primary outcome, was calculated for each participant from the area under the curve of the individual WBA values measured up to 8 hours determined using the trapezoidal method. For the purposes of this calculation, the individual WBA values were converted to a rate of kill per hour (assuming that this rate was constant over the 72 hour period of each WBA culture). The time factors cancel in the calculation of area under the curve, so WBA_cum_ is expressed as absolute kill by the specified time-point after dosing. For comparison of bactericidal activity of TB drug alone and TB drug with celecoxib, WBA_max_ and WBA_cum_ were evaluated using a two-sided paired-sample t-test. Secondary WBA analysis was performed comparing rifampicin or pyrazinamide alone and in combination with celecoxib.

### Ethical and regulatory approvals

The study is registered in clinicaltrials.gov (NCT02602509, date of registration: November 11, 2015) and was approved by the National Healthcare Group’s Domain Specific Review Board (NHG-DSRB) and Health Sciences Authority (HSA), Singapore. All participants provided written informed consent and all research was performed in accordance with the relevant guidelines and regulations.

## Data Availability

The datasets generated and/or analysed during the current study are available from the corresponding author on reasonable request.
